# Increased mechanical loading through controlled swimming exercise induces bone formation and mineralization in adult zebrafish

**DOI:** 10.1038/s41598-018-21776-1

**Published:** 2018-02-26

**Authors:** Santiago Suniaga, Tim Rolvien, Annika vom Scheidt, Imke A. K. Fiedler, Hrishikesh A. Bale, Ann Huysseune, P. Eckhard Witten, Michael Amling, Björn Busse

**Affiliations:** 1 Department of Osteology and Biomechanics, University Medical Center Hamburg-Eppendorf, 22529 Hamburg, Germany; 2 Carl Zeiss X-ray Microscopy, Pleasanton, CA 94588 USA; 3 Department of Biology, Ghent University, 9000 Gent, Belgium; 4 Materials Sciences Division, Lawrence Berkeley National Laboratory, Berkeley, CA 94720 USA

## Abstract

Exercise promotes gain in bone mass through adaptive responses of the vertebrate skeleton. This mechanism counteracts age- and disease-related skeletal degradation, but remains to be fully understood. In life sciences, zebrafish emerged as a vertebrate model that can provide new insights into the complex mechanisms governing bone quality. To test the hypothesis that musculoskeletal exercise induces bone adaptation in adult zebrafish and to characterize bone reorganization, animals were subjected to increased physical exercise for four weeks in a swim tunnel experiment. Cellular, structural and compositional changes of loaded vertebrae were quantified using integrated high-resolution analyses. Exercise triggered rapid bone adaptation with substantial increases in bone-forming osteoblasts, bone volume and mineralization. Clearly, modeling processes in zebrafish bone resemble processes in human bone. This study highlights how exercise experiments in adult zebrafish foster in-depth insight into aging-related bone diseases and can thus catalyze the search for appropriate prevention and new treatment options.

## Introduction

Vertebrate bone is a dynamic organ system that displays extraordinary mechanical properties resulting from its complex hierarchical structure^1,2^. The major functions of the skeleton are load-bearing related to motion, protection of internal organs and maintenance of mineral homeostasis^3^.

Through the essential processes of *bone remodeling* and *modelin*g, optimal skeletal levels of calcium and phosphorous persist during most of an individual’s lifespan. On a microscopic scale, skeletal integrity is maintained by *bone remodeling*, the balanced removal of old bone matrix by bone-resorbing osteoclasts and the deposition of new bone tissue by bone-forming osteoblasts^4,5^. While *bone remodeling* is initiated to repair micro-damages in older bone matrices^6,7^, *bone modeling* is attributed to skeletal growth during development and is later activated in response to mechanical loading of bone, leading to bone formation^8,9^. Conversely, a lack of critical loading will induce increased bone resorption^10^. Bone loss is a typical consequence activated during space travel following prolonged time under micro-gravity conditions^11^. In both processes, bone modeling and bone remodeling, osteoblasts and osteoclasts are orchestrated by mechanosensitive osteocytes that are embedded in the bone matrix^12^. Osteocytes represent the most abundant bone cell and are considered to respond to stimuli including fluid flow, mechanical strain and hydrostatic pressure^3,13,14^. A network comprised of nanometer-sized channels interconnects the osteocytes and facilitates communication with osteoblasts and osteoclasts on the bone surface^15^.

Aging and skeletal diseases such as osteoporosis are associated with alterations in bone remodeling leading to an imbalance between bone resorption and bone formation. Eventually, this imbalance leads to a loss of bone mass and an increase in fracture risk^14,16^. Osteoporosis-related bone loss in humans is likely linked to reduced osteocyte numbers and impaired osteocyte function (*i.e*. reduced mechanosensitivity). In the aged human skeleton this leads to a dysregulation of mechanically driven bone formation, *i.e*. dysregulation of the integral part of the bone modeling process^14,17^. In order to maintain lifelong bone health, it is of crucial importance to fully elucidate the mechanisms behind bone remodeling and load-related bone modeling which cause bone’s structural reorganization.

Several clinical studies showed that physical activity promotes bone strength and leads to measurable fracture risk reduction in humans^18,19^. However, the effects of exercise on bone modeling and remodeling in humans with bone loss syndromes, particularly on a microscopic level, remain insufficiently explored. For many years, mice and rats were the predominant animal models used for the study of bone diseases and changes of both loading scenarios and bone remodeling^20^. More recently, also the ray-finned fish species from the infraclass of teleost fish have been used as novel animal models suited for the study of human bone disorders^20,21^. In particular, among the teleost fish, *zebrafish (Danio rerio)*^20,22^ have been identified as a suitable vertebrate model because the skeleton is a highly conserved organ system.

Also in the fields of physiology^23^, genetics^24^, disease^20^, aging^25,26^ and drug discovery^27^ zebrafish are used as a potent model to study human diseases. With special regard to bone pathologies, new genome editing tools such as TALEN and CRISPR/Cas9 enabled the introduction of targeted deficiencies in genes of zebrafish to generate specific skeletal phenotypes that resemble human skeletal diseases. The possibility to analyze osseous phenotypes in transparent early life stages allows new insights into fundamental processes of skeletal development. The capacity of zebrafish for lifelong tooth replacement and for the regeneration of dermal skeletal elements, such as scales and fin rays further increases the value of this model for skeletal research^28^. In addition, zebrafish may also present a phenotype of spinal deformity resembling osteoarthritis^29^.

**Figure 1:**
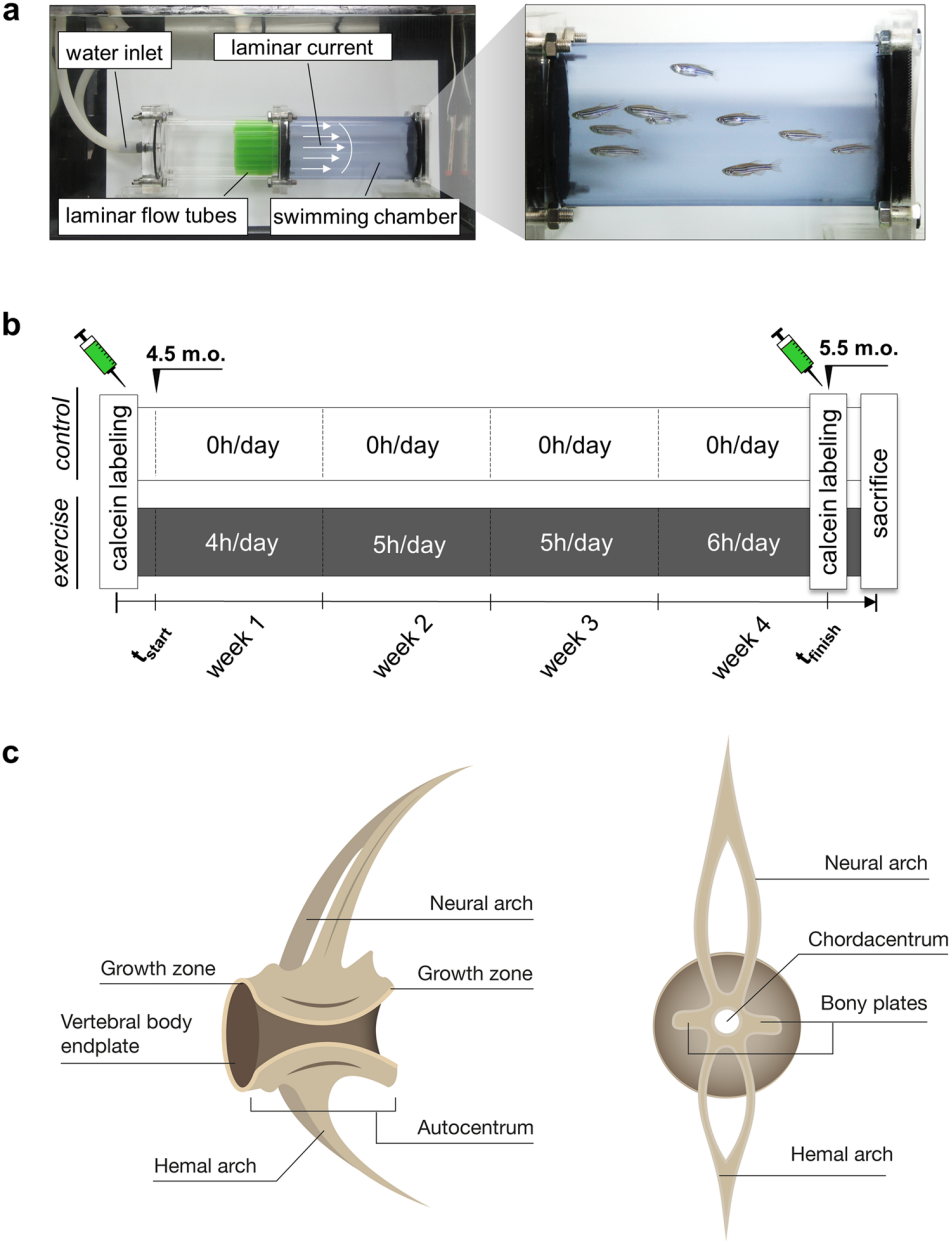
Setup for the experimental musculoskeletal loading of adult zebrafish. (**a**) Swim tunnel used for conducting the exercise experiment. Zebrafish belonging to the exercise group were placed inside the blue chamber during exercise units; white arrows indicate the direction of the water current. Magnified image on the right displays the exercise group during an exercise session. (**b**) Zebrafish belonging to the exercise group were trained for four weeks at step-wise increasing hours per day (4 h/day in week one, 5 h/day in week two and three, 6 h/day in week four) while control zebrafish did not experience any exercise. Both groups were labeled using fluorescent chromophore calcein submersions before and after the experiment. (**c**) Schematic depiction of a zebrafish vertebra. Lateral view (left) and transverse cross section (right).

The vertebrate skeleton is an organ system that adapts and develops in response to mechanical load, to a degree that can override its genetic determination^30^. In this context, Slijper’s goat that adapted its skeleton to bipedal walking is a prominent example^31^, while Zebrafish within the early larval period showed important spatio-temporal changes in skeletogenesis resulting from changes in water flow conditions^32^. To use zebrafish as a model in biomedical research it is thus important to establish the response of the zebrafish skeleton to mechanical load. Adult zebrafish are important study objects since more skeletal diseases in humans arise in adults than in embryos. While zebrafish have been used frequently in developmental biology, the response to potential osteoanabolic stimuli as well as the capability to reflect skeletal adaptations following exercise in a mature zebrafish skeleton remains unknown. Therefore, a controlled experiment targeting the evolving skeletal changes in adult zebrafish is desirable to enable studies on the complex mechanisms of bone loss attributed to aging and sedentary lifestyles. In this context, establishing an experimental loading protocol for adult animals would signify a promising research avenue to identify prevention and treatment options to maintain bone mass (Fig. 1a,b). Despite the anatomical and physiological differences that exist in all established vertebrate models, zebrafish bone displays resemblances to human bone. Interestingly, since adult zebrafish bone also contains osteocytes^33^, its bone structure can provide additional insights into important osteocyte-related skeletal diseases that develop in adult humans, such as the most common bone disease, osteoporosis. A major interest lies in investigating whether osteocytic zebrafish bone reveals similar physiological characteristics and capabilities to adapt to load as the bone of humans and mice.

The anlage of teleost vertebral bodies consists of the mineralized notochord sheath, designated as chordacentrum (Fig. 1c). Bone forms around the mineralized notochord sheath in the shape of a horizontally orientated hourglass, designated as the autocentrum^34^. The anterior and posterior surfaces of the autocentrum constitute the funnel shaped vertebral body end plates. The outer rim of the end plate is the vertebral body growth zone^35^. Bony plates are horizontally attached to the outer contour of the autocentrum, connecting both end plates. Neural and hemal arches insert with basidorsals and basiventrals. Teleost vertebral body malformations typically affect the autocentrum and its outer rim, the growth zone^36,37^. Consequently, this study focuses on the analysis of the autocentrum and the vertebral body end plates. It is hypothesized, that subjecting zebrafish to physical exercise enables the direct observation of the bones’ adaptation to loading. Analyzing how zebrafish bone responds to mechanical load bears great potential for gaining new insights in the field of musculoskeletal research, particularly for identifying potential therapeutic drug targets. The aim of this study is to quantify multiple cellular, compositional, and structural adaptations of zebrafish bone in response to increased physical exercise, thereby elucidating the processes that regulate bone modeling in zebrafish.

## Results

Following 4 weeks of exercise in a swim tunnel for up to six hours on five days per week, exercised zebrafish had larger vertebral autocentrum volumes. Likewise, in exercised zebrafish, bone formation was higher and accompanied by higher bone mineral density in comparison to the control zebrafish.

### Bone microstructural changes

**Figure 2:**
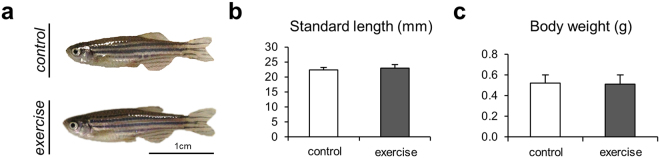
Changes to the body size of the zebrafish. (**a**) Two representative images of a zebrafish from the control group and the exercise group. (**b**) Zebrafish standard length and (**c**) body weight measurements performed after the exercise experiment yielded no significant difference between the groups.

Micro-computed tomography revealed morphological differences between zebrafish vertebrae subjected to exercise and non-exercised controls, even though there were no significant macroscopic differences regarding standard length (Fig. 2a,b) and total body weight between the individuals from the two groups (Fig. 2c).

**Figure 3:**
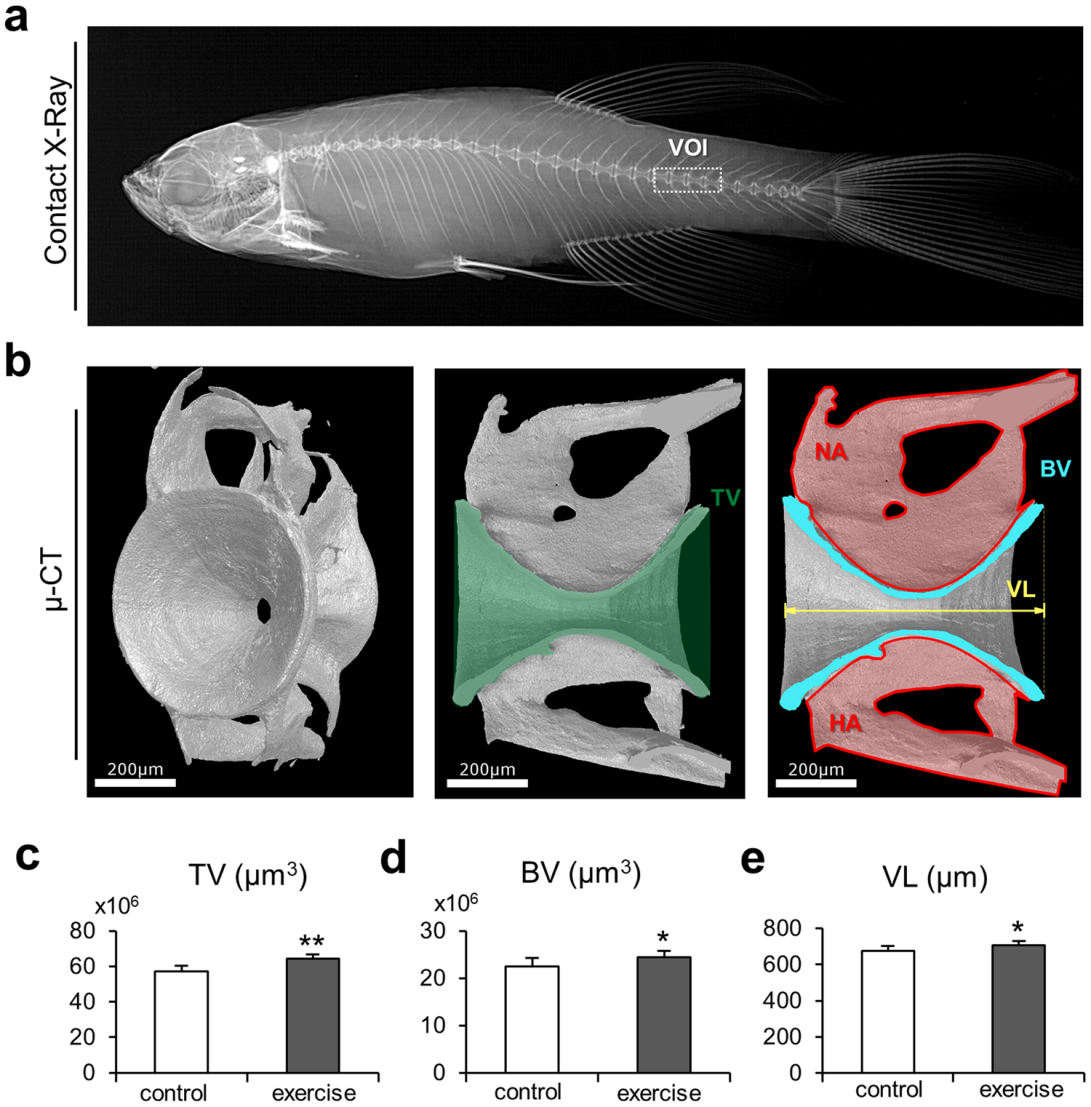
Bone microstructural changes due to musculoskeletal exercise. (**a)** Contact X-ray image displays the volume of interest (VOI) in the caudal spine of zebrafish. All parameters were extracted from caudal vertebrae. (**b**) Micro-CT images display the typical hourglass shape of the autocentrum that represents the core of the zebrafish vertebral body. The volume parameters extracted to quantify skeletal changes due to the swimming exercise are displayed on the sagittal cross-sections of the vertebrae: tissue volume (TV), bone volume (BV), and vertebral length (VL). The neural arch (NA) and the hemal arch (HA) were not included in the quantification of structural parameters via micro-CT. (**c**) Tissue volume was significantly higher in the exercise group. (**d**) Bone volume of the vertebral body was significantly greater in the exercise group. (**e**) Vertebral length increased substantially in the exercise group. The larger bone anatomy of the vertebral bodies of the zebrafish exposed to exercise indicates an increase in bone mass due to increased swimming activity.

The 3D analyses of microstructural differences assessed within the volume of interest in the caudal spine (Fig. 3a,b) showed that the exercise group compared to the control group had a higher tissue volume (64.2 ± 2.6 mio. µm^3^ vs. 57.1 ± 3.2 mio. µm^3^, p < 0.001), a higher bone volume (24.5 ± 1.2 mio. µm^3^ vs. 22.5 ± 1.8 mio. µm^3^, p = 0.016) as well as longer vertebrae (706.9 ± 24.5 µm vs. 673.8 ± 29.2 µm, p = 0.022, Fig. 3c–e).

### Bone modeling characteristics

**Figure 4:**
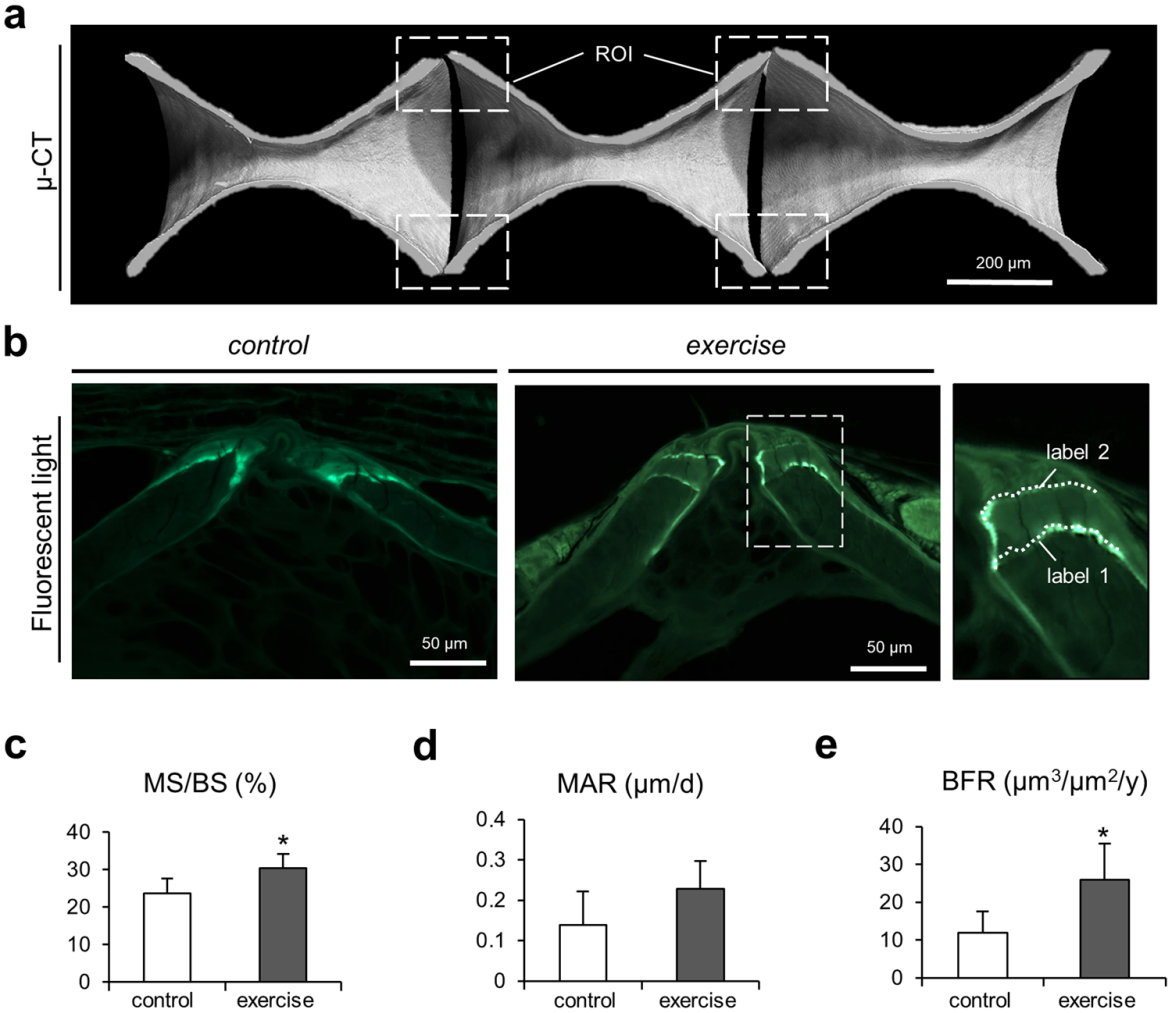
Characterization of bone formation by dynamic histomorphometry. (**a**) Micro-CT images of caudal vertebral bodies display the regions of interest (ROI) including the vertebral body end plates. (**b**) Fluorescence microscopy reveals fluorescent labeling in the end plates from both zebrafish groups. Larger inter-label distance can be observed in exercised zebrafish indicating higher bone formation activity. (**c**) Mineralized surface per bone surface (MS/BS) was significantly higher in the exercise group. (**d**) Mineral apposition rate (MAR) did not vary significantly between groups. (**e**) Bone formation rate (BFR) was significantly higher in exercised zebrafish. The increased deposition of new bone in the end plates of exercised zebrafish explains the increased vertebral length, bone volume and tissue volume measured using micro-CT (see Fig. 3).

Calcein double labeling allowed the detection of areas that showed increased bone formation. *De novo* bone formation occurred in vertebral body end plates (Fig. 4a). The investigated end plates showed more regions with overlapping, non-distinguishable double labels in the non-exercised group. In contrast, the exercised group displayed double labels with larger interlabel distances, indicative of increased bone formation (Fig. 4b). Increased bone formation was most notably observed in posterior caudal vertebrae. The quantification of dynamic histomorphometric parameters yielded a significantly higher mineralizing surface per bone surface (MS/BS, percent of bone surface that displays a calcein label and reflecting active mineralization) in the exercised group than in the control group (30.38 ± 3.86% vs. 23.63 ± 4.07%, p = 0.04) as depicted in Fig. 4c. Although there was no significant difference regarding mineral apposition rate (MAR, rate of new bone deposition) between the groups (Fig. 4d), the bone formation rate (BFR, amount of new bone formed in unit time per unit of bone surface) was 46% higher in the exercise group compared to the control group (25.88 ± 9.65 µm^3^/µm^2^/y vs. 11.93 ± 4.07 µm^3^/µm^2^/y, p = 0.038, Fig. 4e).

### Osteoblastic bone formation

**Figure 5:**
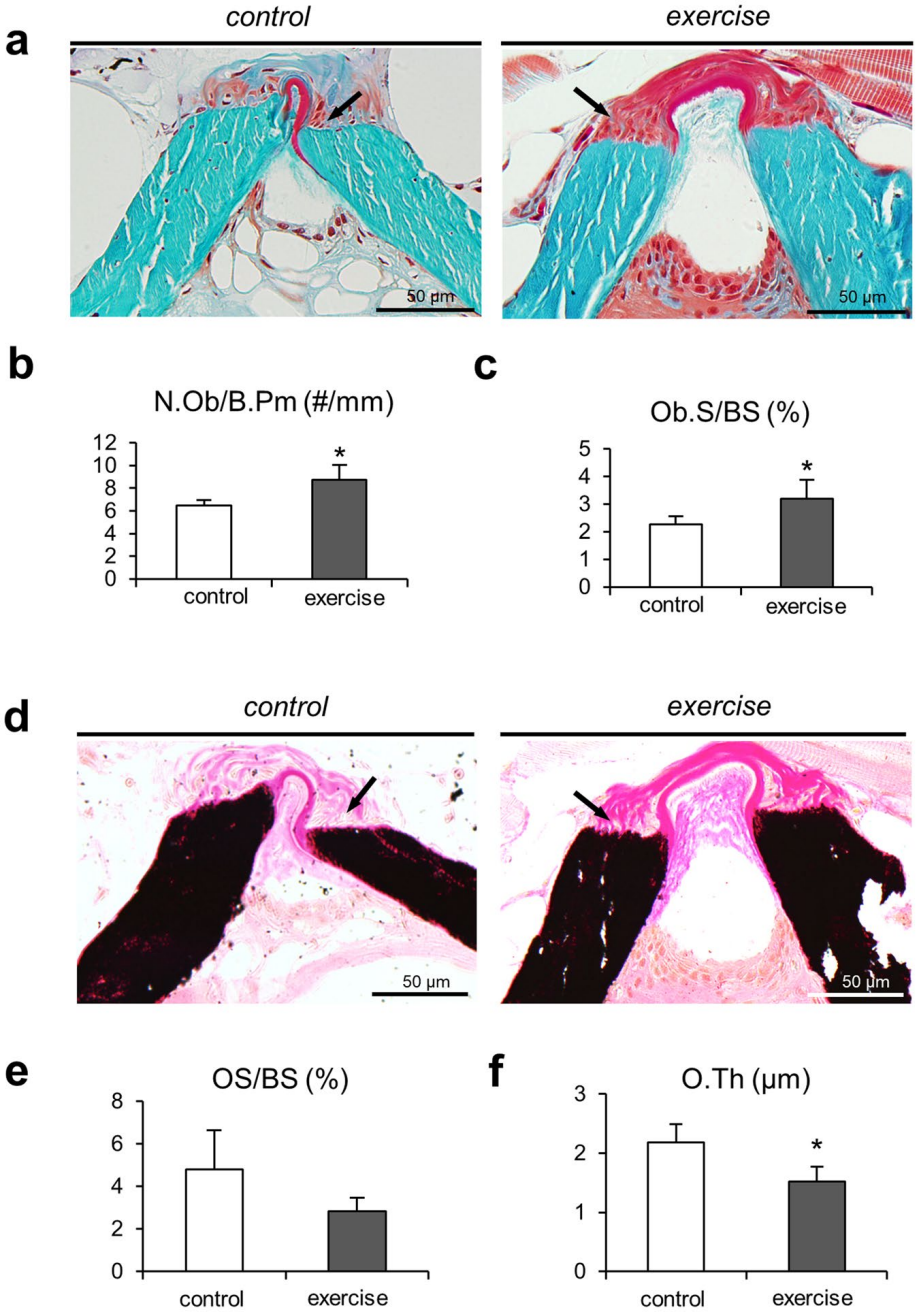
Bone formation mechanism. Static histomorphometric analysis of the bone formation mechanism in zebrafish. (**a**) Masson-Goldner trichrome-stained sections show the end plates of two adjacent vertebrae. Numerous osteoblasts (black arrows) can be observed in the same regions where fluorescence microscopy revealed increased bone formation. (**b**) Quantification of osteoblasts performed on Masson-Goldner trichrome-stained sections yielded higher osteoblast numbers and (**c**) greater osteoblast surface per bone surface in zebrafish from the exercise group. These results are consistent with higher bone deposition rates and higher bone volume found in the exercise group, given that pronounced osteoblast activity is associated with the deposition of new bone material. (**d**) Von Kossa/van Gieson stained sections display non-mineralized bone matrix, *i.e*. osteoid (black arrows), predominantly on the vertebral edges. (**e**) Osteoid surface per bone surface (OS/BS) remained similar between the groups. (**f**) Osteoid thickness (O.Th) was significantly lower in the exercise group compared to controls.

By comparison, the parameters describing the osteoblast activity, number of osteoblasts per bone perimeter (N.Ob/B.Pm) (8.73 ± 1.31 #/mm vs. 6.45 ± 0.49 #/mm, p = 0.02) and osteoblast surface per bone surface (Ob.S/BS) (3.2 ± 0.68% vs. 2.27 ± 0.3%, p = 0.04), were significantly higher in the exercise group, showing a higher bone deposition rate induced by musculoskeletal exercise (Fig. 5a–c). Osteoid surface per bone surface (OS/BS) was not significantly different between the groups and osteoid thickness (O.Th) was significantly lower in the exercise group compared to controls (1.52 ± 0.26 µm vs. 2.18 ± 0.3 µm, p = 0.02, Fig. 5d–f), pointing towards an alteration in the mineralization pattern.

### Osteocyte characteristics

**Figure 6:**
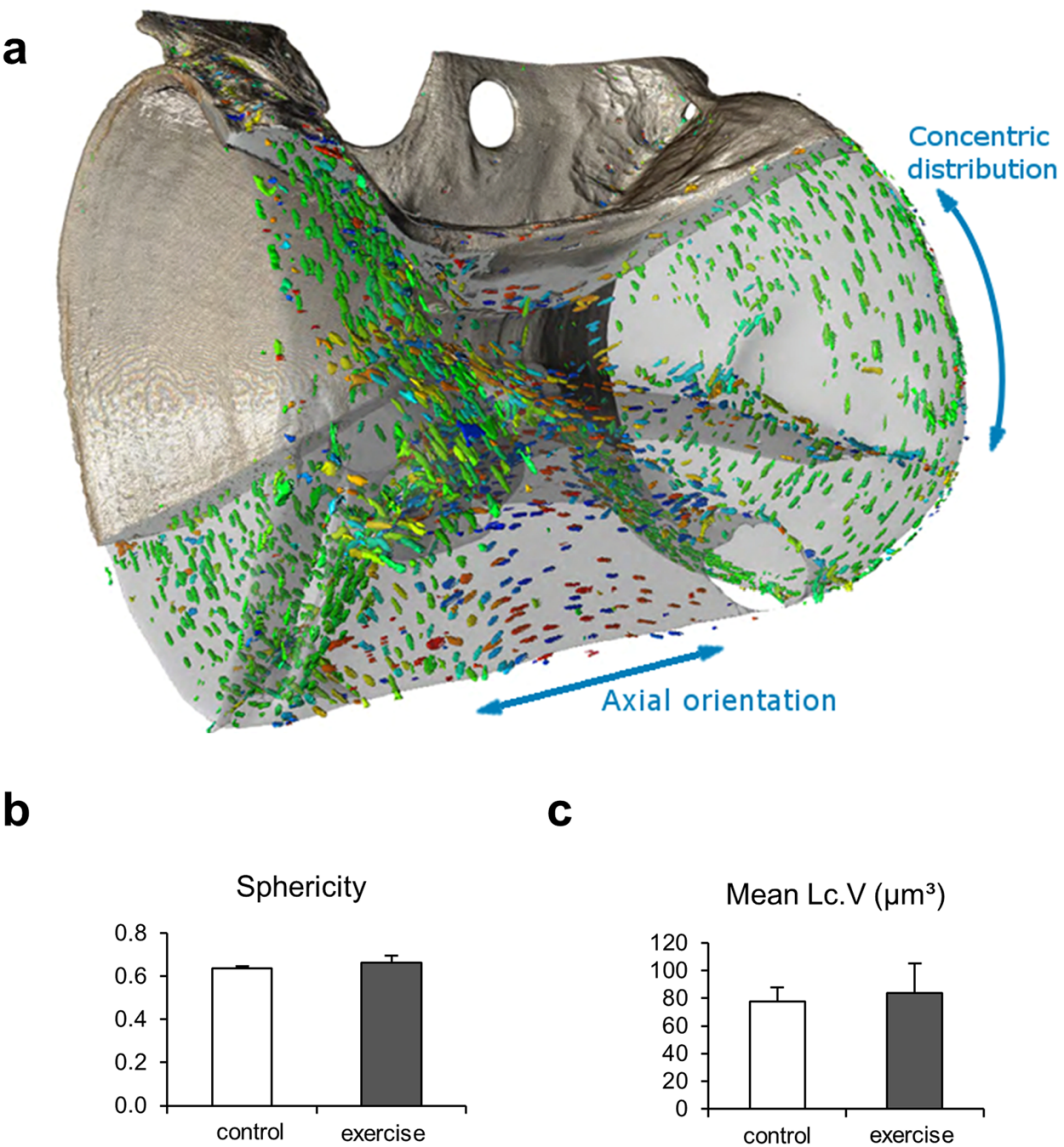
Osteocyte characteristics in terms of osteocyte size, shape and orientation in zebrafish vertebrae. (**a**) Osteocyte lacunae appear ellipsoidal and their distribution and orientation change in relation to the anatomical site. Towards the end regions of the vertebrae, the longitudinal axis of the osteocyte orientation is tangential to the circumference and perpendicular to the long axis of the vertebra. Osteocyte lacunar orientation in the central region of the vertebrae was aligned (parallel) to the long axis of the vertebral body. (**b**) Mean lacunar sphericity and (**c**) mean osteocyte lacunar volume were not significantly different between groups.

3D X-ray microscopy (3D XRM) was used to depict the zebrafish vertebrae in 3D and at high resolution and to quantify osteocyte characteristics at an adequate resolution (0.7 µm) (Supplemental Video 1). In both groups, the analysis showed that the ellipsoidal osteocyte lacunae had a distinct orientation within the zebrafish vertebrae (Fig. 6a). In the vertebral body end plates, the long axes of lacunae were orientated tangential to the circumference, while in the central region of the autocentrum the long axis of the lacunae was aligned with the long axis of the vertebral body. Vertebrae of the exercise and the control group were similar with regard to the lacunar sphericity (0.66 ± 0.03 vs. 0.64 ± 0.01, Fig. 6b) and the mean lacunar volume (83.6 ± 21.8 µm³ vs. 77.6 ± 10.3 µm³, Fig. 6c).

### Bone mineral density distribution

**Figure 7:**
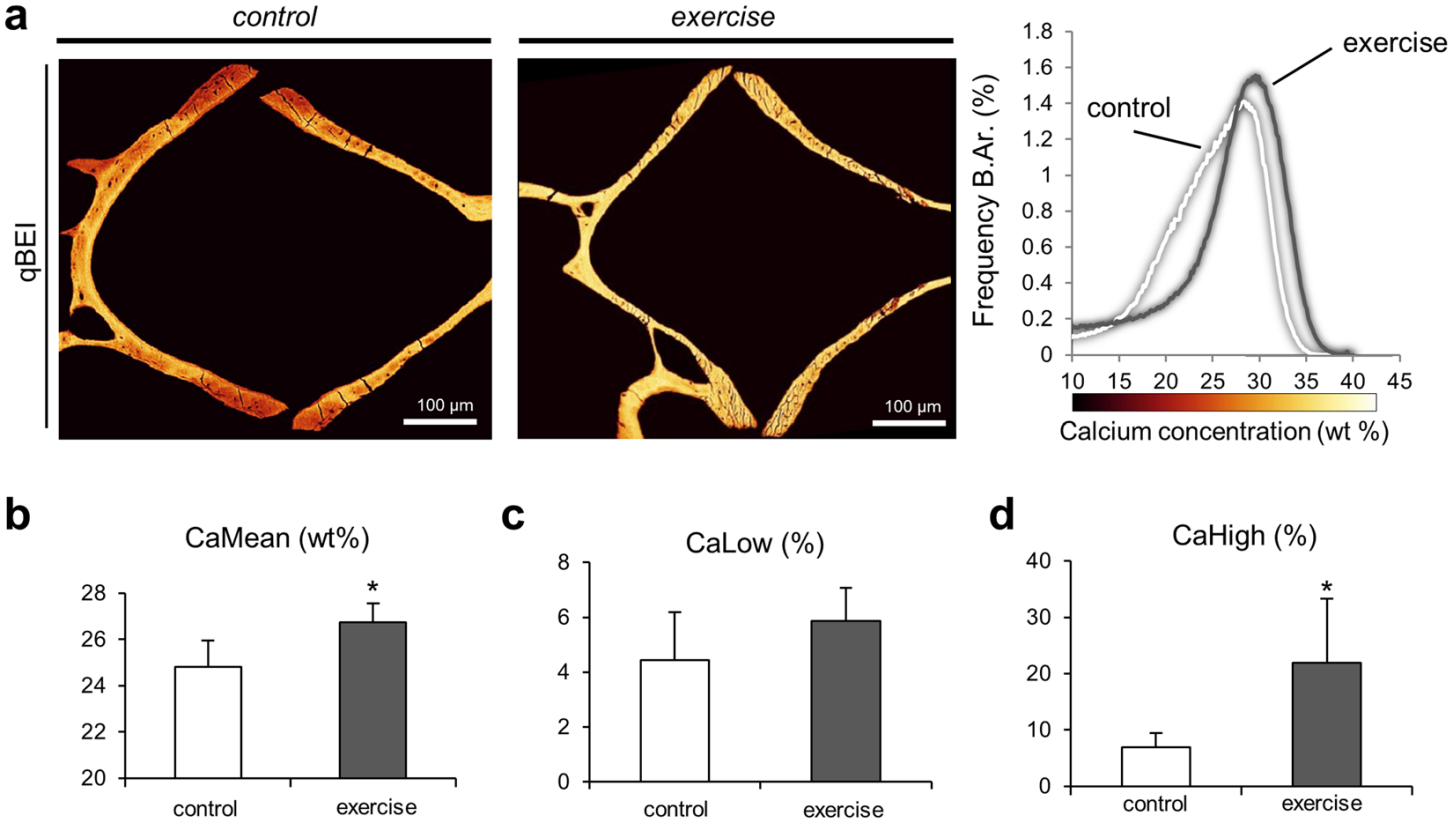
Bone mineral density distribution in zebrafish bone. (**a**) Quantitative backscattered electron microscopy images (pseudo-colored) of zebrafish vertebrae from the control group (left) and the exercise group (center) indicate a higher degree of bone mineralization in exercised zebrafish. Histograms depicting the bone mineral density distribution in calcium weight percentages (right) yielded substantial differences between the groups. (**b**) Exercised zebrafish displayed higher mean calcium content (CaMean) than zebrafish from the control group. (**c**) The amount of bone with a calcium concentration below the 5% threshold (CaLow) remained unchanged between the groups. (**d**) The amount of bone mineralized above the 95% threshold (CaHigh) was significantly higher in the exercise group thus indicating an enhancement of bone mineralization in the exercise group.

The bone mineral density distribution (BMDD) analysis by quantitative backscattered electron imaging (qBEI) is a compositional analysis enabling the measurement of the degree of bone mineralization based on the average atomic number of the material^16,38–40^. In addition to a higher bone mass in zebrafish in the exercised group, as evidenced by *micro-CT* and dynamic histomorphometry, the swimming exercise essentially resulted in a higher degree of bone mineralization (Fig. 7a). The histograms that show the calcium weight percentages in bone display a shift from lower mineralized bone in the control group to higher mineralized bone in the exercise group. This change is expressed by significantly higher mean calcium content (CaMean) in the exercise group compared to controls (26.74 ± 0.83 wt% vs. 24.82 ± 1.13 wt%, p = 0.03, Fig. 7b). While the amount of lower mineralized bone (CaLow) did not differ between the groups (Fig. 7c), the amount of highly mineralized bone (CaHigh) was larger in the exercise group (21.97 ± 11.29% vs. 6.96 ± 2.53%, p = 0.04, Fig. 7d).

## Discussion

Utilizing high-resolution multi-scale analyses enabled the visualization and quantification of processes of bone adaptation in response to musculoskeletal exercise in zebrafish and allowed to assess their potential as an attractive animal model for bone research.

The data provides important insight into the impact of swimming exercise on zebrafish bone. In particular, it is shown that exercise triggers bone adaptation in response to loading. Interestingly, musculoskeletal exercise did not only induce a higher rate of bone formation, but also resulted in a higher degree of bone mineralization. These findings are comparable with results obtained from exercise experiments on larger fish species, which showed a higher mineral content in exercised rainbow trout (*Oncorhynchus mykiss*) and Atlantic salmon (*Salmo salar*)^41,42^. Increased swimming activity in zebrafish has previously been associated with muscle growth and muscle growth marker gene expression^43,44^ as well as with improvement of swimming performance over a certain timespan^25^. In addition to confirming the initial hypothesis, the results of our study show that zebrafish vertebral bodies, similar to human vertebral bodies, have a comparable capability of adapting to increased mechanical load despite their anatomical and developmental differences.

**Figure 8:**
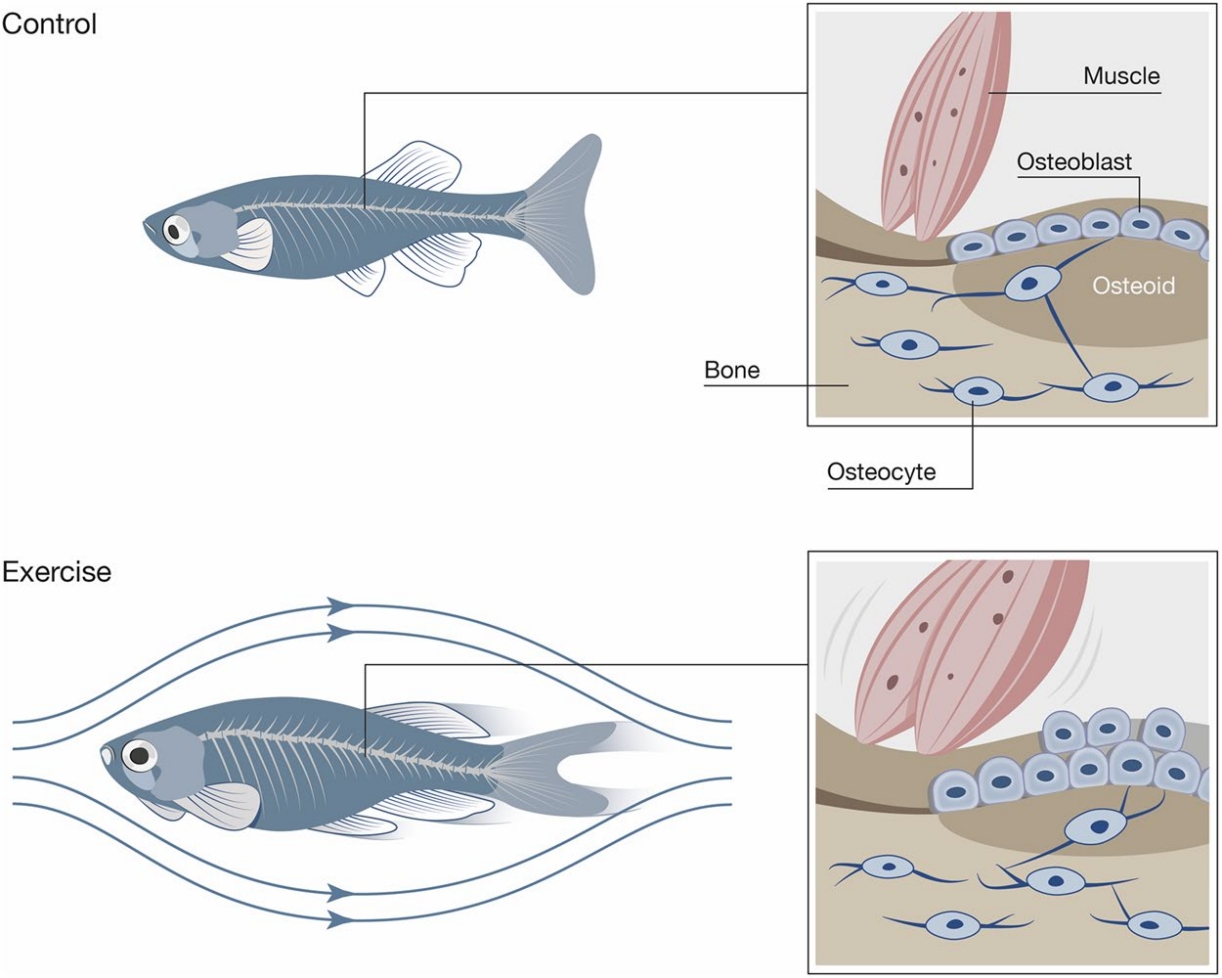
Effects of exercise on bone quality in zebrafish. Schematic depiction of the sum of effects induced by musculoskeletal loading of zebrafish following the swim tunnel experiment. Zebrafish from the exercise group (bottom) were subjected to higher muscular forces that translated into a higher load on zebrafish bone. Higher forces acted as a stimulant for osteoblast activity, which led to an increase of bone formation and mineralization. As a result, exercised zebrafish displayed higher bone mass with a higher degree of bone mineralization when compared to the control group. In humans, increased physical activity^45,46^ and higher bone mineralization is positively correlated with improved bone quality^16^. The results obtained from zebrafish exercise experiments provide therefore deeper insight into the complex mechanisms governing mechanical sensitivity of bone and the pathways that control bone formation and mineralization, thus affecting bone quality.

In humans, bone formation and mineralization relies on the interaction between muscle forces and mechanosensitive osteocytes^13^. Osteocytes detect mechanical strain in the bone tissue, which is induced by muscle forces. Our experimental setup allowed the investigation of this mechanism in zebrafish by exposing the animals to musculoskeletal exercise that triggered new bone formation and increased mineralization as shown in detail in Fig. 8. It has been observed in humans that physical activity such as weight-lifting or practicing a wide range of sports has a beneficial influence on bone quality^24^, particularly on bone mineral density (BMD)^45,46^. The level of bone adaptation in humans obviously depends on the type of loading and appears to be limited to the regions that are exposed to the highest stresses^9,45,47^. Due to ethical considerations exercise studies in humans rely primarily on BMD measurements by dual-energy X-ray absorptiometry (DXA) and blood serum levels^48^. BMD measurements provide, however, only limited and not differentiated information about bone composition and bone structure in age-related deteriorations of bone quality^49–51^. The insufficient data on the influence of mechanical loading on human bone quality and fracture risk highlights the need for animal models suited for comprehensive exercise experiments.

In contrast to humans^14,15^, zebrafish display a reduced lacuno-canalicular network^52^. In tetrapods this network is believed to be essential for the osteocyte-driven bone remodeling. The results of this study indicate that despite the lower osteocyte connectivity observed in zebrafish, increased bone formation was achieved as a result of loading. When osteocyte processes are scarce or absent, Witten & Hall suggest that osteoblasts and bone lining cells could function as alternative receptors for mechanical load^26^. Indeed, various experiments present evidence that morphology, structure and patterns of gene expression change in the jaw bones of advanced teleost species that have no osteocytes in response to altered mechanical load^53,54^. The question of how load is specifically translated into a tissue response in advanced teleosts with anosteocytic bone remains unanswered. The question is especially relevant in the context of early developmental stages as all teleost including zebrafish skeletons are initially devoid of osteocytes^55^. In this context, Fiaz and collagues suggested the possibility of the notochord as a mechanosensor^56^. Some teleost species with anosteocytic bone display cells on the bone surface that extend cytoplasmic cell processes into the matrix similar to mechanosensing osteocytes. Meunier describes this skeletal tissue as primary canaliculated bone in the Spangled emperor fish *Lethrinus nebulosus* as a representative of this type of anosteocytic bone^57^. In mammals, Vatsa *et al*. showed that osteoblastic and osteocytic cells *in vitro* are sensitive to mechanical load^58^. Likewise, mammalian odontoblasts are considered to have sensory capacity^59^. Zebrafish, which display a less developed lacuno-canalicular system, likely rely on similar complementary sensory pathways that do not involve osteocytes. The involvement of osteocytes regarding this aspect can be tested by comparing our results with adaptions to biomechanical load in the species medaka (*i.e.* advanced teleost with similar morphology but anosteocytic bone).

As hypothesized, bone formation in the exercise group occurred most prominently in the vertebral body end plates. Here, the higher number of osteoblasts and their larger size indicate greater osteoblastic activity^5^ and offer an explanation for the observed increase in bone formation. Zebrafish from the control group displayed in the same region fewer osteoblasts, indicative of less bone formation^5^. The musculoskeletal exercise of zebrafish appears to have enhanced the forces acting on the vertebrae and induced bone formation. Regions that experienced the highest bone formation activity, in this case the end plates of the vertebral bodies, most likely indicate regions that experienced the highest stress concentration. In humans, a similar behavior can be observed as regions of bone that experience higher stress also display higher bone formation and higher osteoblast activity^10,45^.

Key aspects of bone modeling such as resorption lacunae and multinucleated osteoclasts can be observed in the zebrafish skull^33,55^. Different from teleost hemal and neural arches that require bone resorption of the endosteal bone surface for extension of the lumina, the vertebral body endplates grow and extend by periosteal bone apposition only; osteoclastic resorption is not required for normal development^60^. In adult zebrafish vertebrae, we were able to observe how the orientation of osteocyte lacunae changes in different regions of the vertebral bodies. In the central region, osteocyte lacunae are oriented parallel to the long axis of the vertebrae while in the region of the vertebral body end plates, osteocyte lacunae are oriented circumferentially within the vertebra. This specific arrangement of osteocyte lacunae found in the zebrafish vertebrae does not only indicate the orientation of the collagen^61^ but also signifies the direction of the main forces experienced by the vertebrae^62^. Osteocyte lacunae represent also volumes with a low modulus inside a material and thus are expected to produce stress concentrations around them^62^. This fact explains the tendency of osteocyte lacunae to be oriented parallel to the forces acting on bone in order to reduce the stress concentrations surrounding osteocyte lacunae^62^. The orientation of the long axis of the osteocyte lacunae therefore likely represents the direction in which zebrafish vertebrae experience the highest stress. During early developmental stages, the stresses acting upon the vertebrae may explain the change in orientation of osteocyte lacunae as the vertebral diameter increases. Initially, the main forces acting upon the vertebrae should possess an axial orientation contributing therefore to the axial orientation of osteocyte lacunae. As the vertebrae grow in diameter, more complex stress arrangements must be accounted for, leading to a circumferential arrangement of osteocyte lacunae.

It is interesting to note, that new bone formation is exclusively induced in regions of higher stress concentration, while at the same time bone mineral density is increasing throughout the entire vertebral body. In humans, newly formed bone typically displays a lower mineral density than old bone. Following primary mineralization, secondary mineralization leads to further increases in bone mineral content, which correlates with tissue age^63^. The difference in bone mineral density distribution measured between groups indicates that the musculoskeletal exercise induced substantial increases in calcium content of old and newly formed bone in a relatively short period of time. Additionally, the higher degree of mineralization (*i.e.* calcium content) of the entire zebrafish vertebra is not restricted to the vertebral bone tissue but also affects the mineralized notochord sheath. This supports the notion that increases in mineral content can be achieved without the contribution of osteocytes as the notochord sheath is completely devoid of mechanosensing osteocytes. Bone mineralization in humans is clearly correlated with fracture resistance^50,64,65^, which implies that the higher bone mineralization observed in the zebrafish exercise group likely represents an improvement of the mechanical properties of bone.

In conclusion, the results of this study demonstrate that musculoskeletal exercise of zebrafish is a novel investigative approach that can promote new research in the fields of bone regeneration and age-related bone diseases. Utilizing zebrafish as model has a few limitations: Zebrafish have no osteons as it is known from human load-bearing cortical bone and zebrafish have a swim bladder, which changes the experienced mechanical loading during swimming in comparison to tetrapods. Further on, zebrafish live in environmental conditions that differ substantially from humans (*i.e.*, aqueous ionic concentrations, temperature of the water (≈28 °C)).

The here reported exercise regimen can be applied to genetically modified zebrafish strains with skeletal phenotypes, which resemble common metabolic bone pathologies including osteoporosis^66^ or rare genetic disorders including osteogenesis imperfecta^67^. Musculoskeletal exercise of zebrafish not only offers the chance to investigate the impact of exercise on the progression of these diseases, but also the chance to determine the efficacy of new or existing treatments when combined with musculoskeletal exercise. Interestingly, zebrafish show impaired swimming performance with advanced age^42^, indicating that the zebrafish is an extremely useful candidate to study aging-related bone health in combination with musculoskeletal exercise. Specifically, with regard to the similarities in essential bone reorganization mechanisms between humans and teleosts, zebrafish qualify as a valuable and promising model for future mechanistic research in the field of osteology.

## Methods

To test the increased skeletal loading on bone in zebrafish, we designed a musculoskeletal exercise regimen. After completion of the exercise regimen, the caudal vertebrae were compared using high-resolution imaging methods in order to detect exercise-induced bone changes. All bone histomorphometric measures were determined considering the ASBMR nomenclature^68^.

### Animal models

We acquired 20 zebrafish (*Danio rerio*) of four and half months of age and divided them into a control group and an exercise group (n = 10 per group). Each group was placed in a 60 l water tank with identical water conditions regarding temperature (26 °C), pH (7.4), water hardness (6–12°dH), nitrite (<0.3 mg/L) and nitrate (25–100 mg/L) levels. All zebrafish were fed flake food twice a day until satiation and were kept on a 14/10 hours light/dark photoperiod. The experiment gained prior approval by the institutional review board (IRB) of the University Medical Center Hamburg-Eppendorf (UKE) and the ethics committee of the city of Hamburg (No. 42/16), and the methods were performed in accordance with the approved guidelines.

### Experimental setup and sample preparation

A third 70 l water tank containing a custom-built swim tunnel was used to exercise the zebrafish (Fig. 1a). The swim tunnel was adapted with a stack of flow tubes to maintain a continuous laminar flow. The swimming exercise regimen consisted of five days of exercise per week for a period of four weeks. Zebrafish from the exercise group were coerced to swim against the flow and moved inside the swim tunnel in a natural motion pattern throughout the exercise as shown in Fig. 1a and Supplementary Video 2. Flow velocity in the swim tunnel was kept constant at 12 cm/s while the number of exercise hours increased from four hours per day during week one, to six hours per day during week four. The control group remained in a 60 l water tank for the duration of the experiment and was not subjected to exercise.

To allow the detection of dynamic bone formation, zebrafish were labeled using chromophore calcein (Calcein, Sigma-Aldrich Chemie GmbH, Munich Germany) three days before starting the exercise experiment. This procedure was repeated on the last day of the exercise experiment. Calcein labeling was performed using the submersion protocol described by Du *et al*.^69^. Briefly, immersion solutions (0.2%) were prepared by dissolving 2 g of calcein powder in 1 liter of deionized water, subsequently immersing zebrafish for 6 min. All zebrafish were sacrificed three days later using an overdose of tricaine methane sulfonate (MS222, 250 mg/l, Sigma-Aldrich Chemie GmbH, Munich Germany). All zebrafish were measured after the experiment to quantify possible macroscopic body changes induced by the musculoskeletal exercise. Body length was measured as standard length, excluding the length of the caudal fin. Three zebrafish spines per group were dissected for subsequent scanning using micro-CT. The remaining zebrafish were fixed in 3.7% formaldehyde for three days, dehydrated with an increasing alcohol series and embedded in methyl-methacrylate blocks. Samples used for static histomorphometric analysis were cut into 4 µm thick sections while dynamic histomorphometry was performed on 12 µm thick sections. Masson-Goldner trichrome and von Kossa/van Gieson protocols were used for the analysis of bone cells and mineralized hard tissue, respectively^70,71^.

### Micro-computed tomography

Vertebral microstructural differences were assessed using micro-computed tomography (Skyscan 1272, Bruker, Kontich, Belgium) at a resolution of 1 µm. We scanned nine vertebrae per group with 45 kV and 200 µA without an X-ray filter. Ring artifact and beam hardening corrections were kept constant for all samples during reconstruction with NRecon (Bruker, Kontich, Belgium). After applying a fixed threshold for all samples, 3D evaluation was conducted using CTAn (Bruker, Kontich, Belgium). Neural and hemal arches were excluded from the volume analysis. The tissue volume (TV) was defined as the whole volume of a vertebra including the inner open space, whereas the bone volume (BV) was defined as the vertebral body without the inner space. Volume and length (VL) of the vertebral bodies was determined.

### 3D X-Ray microscopy

The osteocyte network in zebrafish vertebral bodies was quantified using a 3D X-ray microscope (ZEISS Xradia 520 Versa, Carl Zeiss X-ray Microscopy, Pleasanton, CA, USA). Zebrafish vertebrae were scanned at a resolution of 0.7 µm and vertebrae were analyzed using 3D evaluation software (Visual SI Advanced, Object Research Systems Inc, Montreal, Canada; ImageJ2 v1.5, https://imagej.nih.gov/ij). Orientation of the osteocyte lacunae in relation to the long and short axis of the vertebral bodies, sphericity (Sph, 0–1) and mean lacunar volume (Lc.V/N.Lc, μm^3^) parameters were imaged with 3D X-ray Microscopy (3DXRM) in a total of six caudal vertebrae from two animals per study group.

### Dynamic and static histomorphometry

To detect and quantify bone formation, calcein double labels were assessed in fluorescent light microscopy. The following parameters were evaluated using OsteoMeasure (OsteoMetrics, Atlanta, GA, USA): The mineralizing surface per bone surface (MS/BS,%), representing active mineralization, calculated as percent of bone surface that displays a calcein label; the mineral apposition rate (MAR, µm/d), representing the rate of new bone deposition, calculated as distance between consecutively deposited double labels divided by the time interval between them; and the bone formation rate (BFR, µm^3^/µm^2^/y), representing the amount of new bone formed, calculated by multiplying mineralizing surface with the mineral apposition rate.

Osteoblast- and osteoid-related parameters were also quantified using the OsteoMeasure histomorphometry system. The parameters - number of osteoblasts per bone perimeter (N.Ob/B.Pr, 1/mm), osteoblast surface per bone surface (Ob.S/BS, %) as well as osteoid indices, including osteoid surface per bone surface (OS/BS, %) and osteoid thickness (O.Th, µm), were extracted in order to quantify cell based bone deposition activity. In each sample section, eight microscopic fields were evaluated at 20-fold magnification. The cells were counted in Masson-Goldner-stained sections, and osteoid indices where evaluated in von Kossa/van Gieson-stained sections.

### Quantitative backscattered electron imaging (qBEI)

Embedded zebrafish specimens were polished to a coplanar finish and carbon coated. The scanning electron microscope (LEO 435 VP; LEO Electron Microscopy Ltd., Cambridge, England) was operated at 20 kV and 680 pA at a constant working distance (BSE Detector, Type 202; K.E. Developments Ltd., Cambridge, England). Bone mineral density distribution (BMDD) analysis was performed as previously described^16^ to assess the degree of mineralization of the vertebral bone (N = 4 per group). A carbon-aluminum standard serves to calibrate the system and consequently allows a quantification of mineral as a calcium weight percentage^49,72^. Recorded parameters were mean calcium content CaMean (wt%), amount of low mineralized bone (CaLow in % bone area, percentage of bone area that is mineralized below the 5th percentile of a defined reference group) and amount of highly mineralized bone (CaHigh in % bone area, percentage of bone area containing Ca concentration above the 95th percentile).

### Statistical Analysis

Statistical analysis was carried using SPSS (Version 24, IBM, Armonk, NY, USA) and using a significance level of 5%. Normal distribution was tested using Shapiro-Wilk tests and homogeneity of variance using Levene’s test. All parameter comparisons between groups were carried out using independent t-tests.

### Data availability statement

The data that support the findings of this study are available from the corresponding author upon reasonable request.

## Electronic supplementary material

Supplemental Video 1

Supplemental Video 2
